# Transcriptional landscapes at the intersection of neuronal apoptosis and substance P-induced survival: exploring pathways and drug targets

**DOI:** 10.1038/cddiscovery.2016.50

**Published:** 2016-08-01

**Authors:** S Paparone, C Severini, M T Ciotti, V D’Agata, P Calissano, S Cavallaro

**Affiliations:** 1 Institute of Neurological Sciences, Italian National Research Council, Via Paolo Gaifami, 18, Catania 95125, Italy; 2 Institute of Cell Biology and Neurobiology, Italian National Research Council, Via del Fosso di Fiorano 64, Roma 00143, Italy; 3 European Brain Research Institute, Via del Fosso di Fiorano 64, Roma 00143, Italy; 4 Department of Biomedical and Biotechnological Sciences, Section of Human Anatomy and Histology, University of Catania, Catania 95125, Italy

## Abstract

A change in the delicate equilibrium between apoptosis and survival regulates the neurons fate during the development of nervous system and its homeostasis in adulthood. Signaling pathways promoting or protecting from apoptosis are activated by multiple signals, including those elicited by neurotrophic factors, and depend upon specific transcriptional programs. To decipher the rescue program induced by substance P (SP) in cerebellar granule neurons, we analyzed their whole-genome expression profiles after induction of apoptosis and treatment with SP. Transcriptional pathways associated with the survival effect of SP included genes encoding for proteins that may act as pharmacological targets. Inhibition of one of these, the *Myc* pro-oncogene by treatment with 10058-F4, reverted in a dose-dependent manner the rescue effect of SP. In addition to elucidate the transcriptional mechanisms at the intersection of neuronal apoptosis and survival, our systems biology-based perspective paves the way towards an innovative pharmacology based on targets downstream of neurotrophic factor receptors.

## Introduction

Neuronal apoptosis and survival are orchestrated by intrinsic transcriptional programs regulating. These programs are activated by multiple extracellular and/or intracellular signals, including the absence or presence of neurotrophic factors. Primary cultures of cerebellar granule neurons (CGNs) represent the election model to examine the mechanisms underlying neuronal apoptosis and survival.^1^ In this *in vitro* paradigm, a rapid apoptotic cell death occurs within 24 h after removal of serum and lowering of extracellular potassium from 25 to 5 mM. Engagement of apoptosis requires transcription and protein synthesis and becomes irreversible after 6 h from induction.^2^ Before this ‘commitment point’ CGNs can be rescued by the activation of specific signal-transduction pathways or by the treatment with specific neurotrophic factors, such as insulin-like growth factor-1 (IGF-1),^2^ pituitary adenylyl cyclase-activating polypeptide (PACAP),^3,4^ gastric inhibitory polypeptide (GIP)^5^ and substance P (SP).^6^ Although their effects are mediated by different receptors and intracellular second messengers, their signaling pathways converge into the nucleus and regulate gene expression.^3,7–9^

The advent of high-throughput technologies is now offering a systems biology-based perspective to analyze the mechanisms underlying neuronal apoptosis and survival. Indeed, the ability of a neuron to promote or evade apoptosis depends on the activity of an integrated network of genes and their encoded proteins, which never work alone but interact with each other in highly structured networks. In recent years, we have begun to explore the systems biology of neuronal apoptosis and survival cross-paths by analyzing whole-genome expression profiles.^1,10,11^ Although our previous studies represent the first glimpse into the transcriptional landscape of neuronal apoptosis and survival, they suggest the existence of a conserved transcriptional program. Indeed, the survival effects of IGF-1 and PACAP share striking similarities and are propagated by common transcriptional cascades.^1,7^ In the present study, we have extended our analysis to SP, a potent antiapoptotic neurotrophic factor, which belongs to the tachykinins neuropeptide family.^12^

## Results

### Induction of apoptosis and rescue by SP

**Figure 1:**
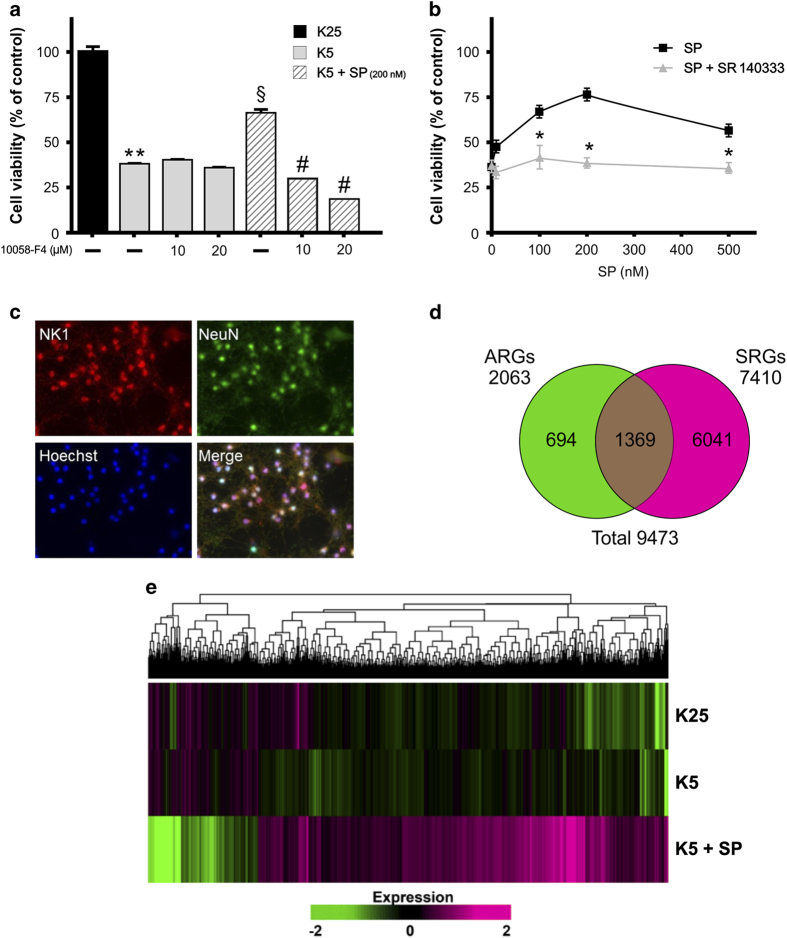
Pharmacological and transcriptional effects of SP following induction of apoptosis in CGNs. (**a**) Effect of SP and Myc inhibitor on CGNs viability. Primary cultures of CGNs at 6 DIV were switched into serum-free medium containing 5 mM KCl for an acute induction of apoptotic death. After 48 h, neuronal viability was assessed by counting the number of intact nuclei. Neuroprotective effects of 200 nM SP administration was reverted by cotreatment with the Myc inhibitor. Values for neuronal viability represent the mean±S.E.M. of four determinations in two different experiments. Statistically significant differences were calculated by one-way analysis of variance (ANOVA) followed by Bonferroni’s test for multiple comparisons (*P<0.05, **P<0.01 *versus* K25; ^§^*P*<0.01 *versus* K5; ^#^*P*<0.01 *versus* SP treatment). (**b**) Neuroprotective effects of SP were inhibited by the NK1-selective receptor antagonist SR 140333 (25 nM). (**c**) Representative immunofluorescence photomicrographs showing CGNs stained with an antibody against NK1 receptor (red) or NeuN to visualize neurons (green). Total nuclei were stained with Hoechst (blue). (**d**) Genes differentially expressed in control (K25) *versus* apoptotic (K5) CGNs were defined as ARGs. Genes differentially expressed in apoptotic (K5) CGNs *versus* those rescued from death by SP treatment (K5+SP) were defined as SRGs. (**e**) Hierarchical clustering. A hierarchical clustering was used to cluster differentially expressed genes in control (K25), apoptotic (K5) and rescued (K5+SP) CGNs. In this two-dimensional presentation, each row represents a single SRG, whereas the columns represent each of the three different experimental conditions analyzed (K25, K5, K5+SP). As shown in the color bar, the red color indicates upregulation and the green color downregulation.

As previously demonstrated, apoptosis of CGNs induced by removal of serum and lowering of extracellular potassium from 25 to 5 mM was antagonized by treatment with SP.^2^ The dose-dependent rescue effect of SP reached its maximal efficacy (75%) at 200 nM and was completely reverted by cotreatment with 25 nM SR 140333, a specific antagonist of neurokinin 1 (NK1) receptor (Figures 1a and b).^13^ The presence of NK1 receptors in NeuN-positive CGNs was confirmed by immunocytochemistry analysis (Figure 1c) and supports the direct neuroprotective action of SP on CGNs.

### Whole-genome expression changes underlying CGN apoptosis and rescue by SP

We characterized whole-genome expression profiles of CGNs 6 h after the induction of apoptosis or its rescue by a maximal effective dose of SP (200 nM). Then, we used two complementary approaches to simultaneously investigate changes of individual genes and functional gene groups.

#### Differentially expressed genes in apoptotic and rescued CGNs

When gene expression profiles of control (K25) and apoptotic (K5) CGNs were compared, 2063 genes, operationally defined as ‘apoptotic related genes’ (ARGs), showed significant changes. By comparing gene expression profiles in CGNs 6 h after the induction of apoptosis (K5) with those of neurons rescued by SP treatment (K5+SP), 7410 genes were found differentially expressed and operationally defined as ‘survival-related genes’ (SRGs) (Figure 1d). Intersection of ARGs and SRGs comprised 1369 genes (Figure 1d). A comprehensive picture of differentially expressed genes is shown in Figure 1e, where ARGs and SRGs (a total of 9473 genes) are grouped on the basis of similarity in their expression patterns with a hierarchical clustering method. The significant impact of apoptosis and survival on the CGN transcriptome can be distinguished by the color matrix.

#### Deregulated pathways in rescued CGNs by SP

A total of 95 statistically significant pathways were identified in the context of SRGs (Supplementary Data S9). To reduce redundancy of deregulated pathways and simplify their comprehension, the most significant variations implicated in these pathways were summarized in Figures 2,3,4,5,6,7,8 and Supplementary Figures 9–15.

### Drug targets discovery

**Figure 2:**
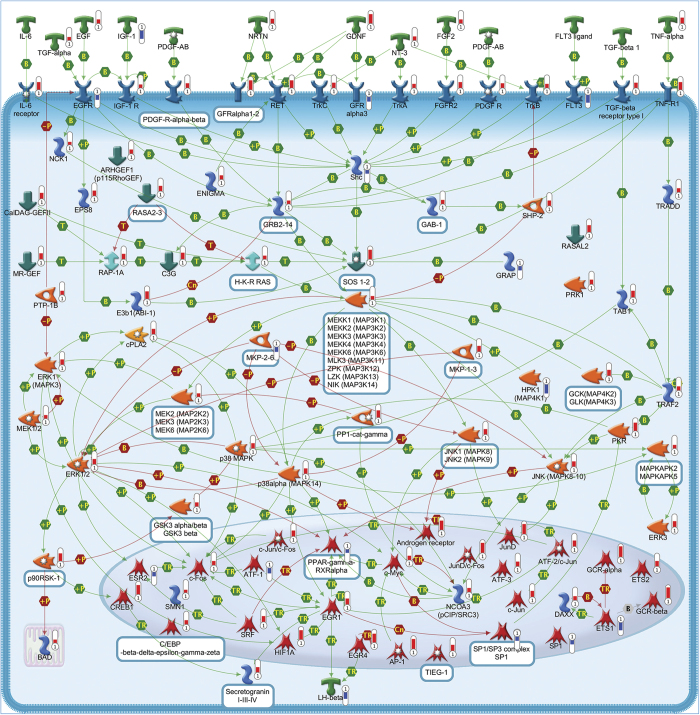
Mitogen-activated protein kinase (MAPK) signaling pathway. The canonical MAPK cascade is activated by a wide variety of stimuli and receptors promoting cell survival and proliferation. This pathway includes a set of adaptors, such as SHC and GRB2, linking the receptor to a guanine nucleotide exchange factor (GEF) such as SOS and C3G, transducing the signal to small GTP-binding proteins (Ras), which in turn activate the core unit of the cascade composed of a MAPKKK (Raf), a MAPKK (MEK1/2 (MAPK/ERK kinase-1/2)) and a MAPK (ERK). An activated ERK dimer, in turn, regulates several targets in the cytosol and translocates to the nucleus where it phosphorylates a variety of transcription factors regulating gene expression. Negative regulators of ERK pathway include MKPs. Pathway objects and links are described separately in the Supplementary Figure 16.

Many of statistically altered genes in CGNs rescued by SP encode for drug targets whose modulation might exert antiapoptotic or prosurvival effects (Supplementary Data S10 and S11). Although the pharmacological exploitation of these targets goes beyond the aim of the present study, we functionally analyzed one of these genes, *Myc*, which is upregulated in rescued *versus* apoptotic neurons (Figure 2). Treatment of CGNs with different concentrations of the selective Myc inhibitor, 10058-F4, reverted in a dose-dependent manner the rescue effect of 200 nM SP (Figure 1a). These data support a direct role of *Myc* in the neuroprotective activity of SP.

## Discussion

Neuroprotective action of SP is associated with profound transcriptional changes involving different functional categories, each of which will be discussed separately in the following paragraphs.

### Signal transduction

CGN rescue by SP involves the different expression of genes encoding for different extracellular mediators and their receptors, as well as numerous downstream proteins of various signaling pathways.

#### Extracellular mediators

##### Neuropeptides

The rescue program induced by SP included the altered expression of several neurotrophic factors, neuropeptides and their receptors notoriously associated with neuroprotective and prosurvival properties (Figures 2, 4 and 6).^14^ Numerous genes encoding for cytokines, chemokines and their corresponding receptors have also showed different expression in rescued CGNs, which are consistent with their role in mediating survival signals.^15^ Moreover, deregulated expression of some interleukins and their receptors is in line with previous studies showing the capacity of these mediators to influence neuronal survival (Figures 2 and 5).^16^

##### Neurotransmitter receptors

**Figure 3:**
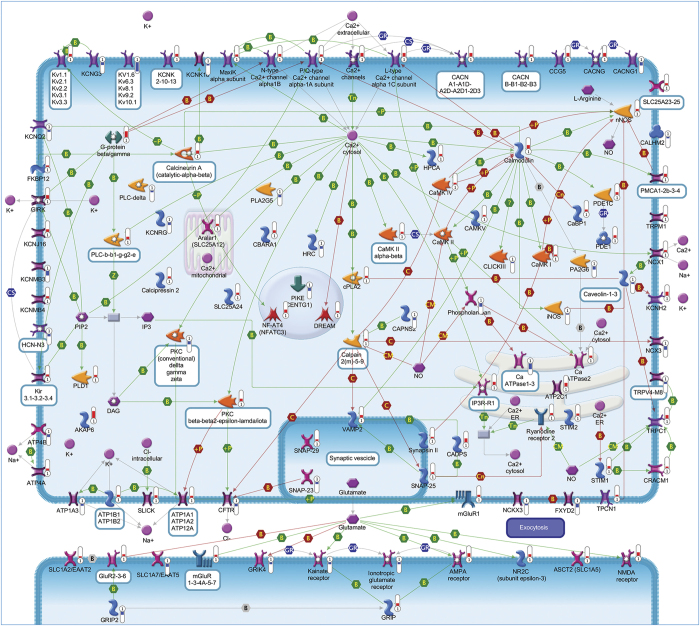
Ion homeostasis. Ca^2+^ homeostasis is regulated by several transporters. Ca^2+^ influx is primarily mediated by voltage-gated Ca^2+^ channels (VGCCs), transient receptor potential channels (TRPs) and ligand-gated channels. Ca^2+^ efflux is achieved by plasma membrane Ca^2+^ ATPases (PMCAs), sodium calcium exchangers (NCXs) or Na^+^/NCKX. Release of Ca^2+^ from the ER/SR is mediated through IP3 or ryanodine receptors. Store-operated channels are activated by stromal interaction molecule 1 (STIM1) protein that senses the store depletion and triggers the opening of the Ca^2+^-release-activated Ca^2+^ channels and calcium release-activated calcium modulators. Store-operated channels entry (SOCE) is also modulated by K^+^-permeable channels, KCa and Kv, which are regulated by intracellular Ca^2+^ and depolarization, respectively. These two channels help sustain SOCE generation by inducing cell hyperpolarization. Additionally, the Na^+^-permeable channel transient receptor potential cation channel subfamily M member (TRPM) is gated by intracellular Ca^2+^ and reduces Ca^2+^ mobilization. The reuptake of Ca^2+^ into the ER/SR is primarily mediated by sarcoplasmic/ER Ca^2+^ ATPase. Pathway objects and links are described separately in Supplementary Figure 16.

The neuroprotective effects of SP were associated with the differential expression of genes encoding for both inhibitory and excitatory neurotransmitter receptors: GABA-A, GABA-B, NMDA, AMPA, kainate and metabotropic glutamate, which are known to profoundly affect neuronal fate (Figure 3 and Supplementary Figure 9).^17,18^ In addition to these, we observed the differential expression of adrenergic, dopaminergic, serotonergic and adenosine receptors, which may further contribute to the fine regulation of neuronal survival (Figure 6).^19–21^

#### Intracellular signaling pathways

**Figure 4:**
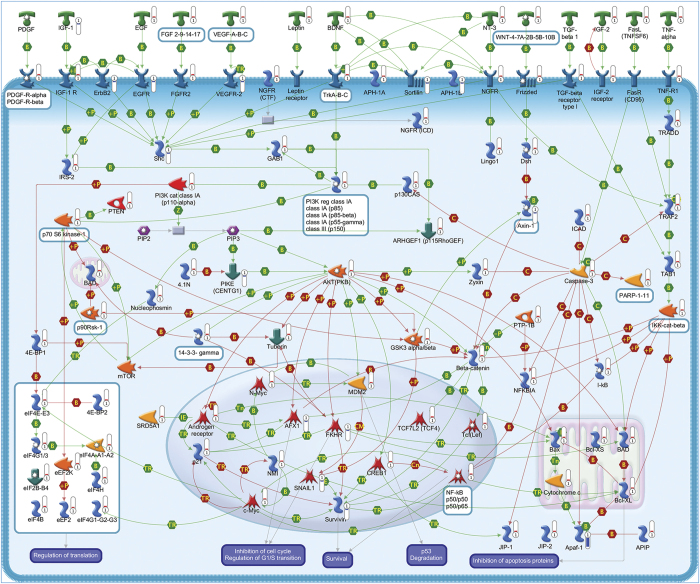
PI3K-AKT (phosphatidylinositol-4,5-bisphosphate 3-kinase-AKT) signaling pathway. The PI3K-PKB/AKT pathway is controlled by a multistep process. Activated receptors directly stimulate class 1A PI3Ks bound via their regulatory subunit or adapter molecules such as the insulin receptor substrate (IRS) proteins. This triggers activation of PI3K and conversion of PIP2 (phosphatidylinositol-4,5-bisphosphate) into PIP3 (phosphatidylinositol3,4,5-trisphosphate). PKB/AKT binds to PIP3 at the plasma membrane, allowing PDK1 to activate PKB/AKT. Physiological roles of AKT include activation of protein synthesis, inhibition of apoptosis, cell cycle progression and transcription factors regulation. These are exerted by phosphorylation of a variety of downstream substrates, including caspase-3, BAX, BAD, Bcl-XL, FKHR, IKK, NF-*κ*B, GSK3, AR, MDM2, CREB, p21, p70S6K1, JIP1 scaffold protein and a series of initiation factors. Dephosphorylation by protein phosphatase-2A (PP2A) and the conversion of PIP3 to PIP2 by PTEN antagonize AKT signaling. Pathway objects and links are described separately in Supplementary Figure 16.

**Figure 5:**
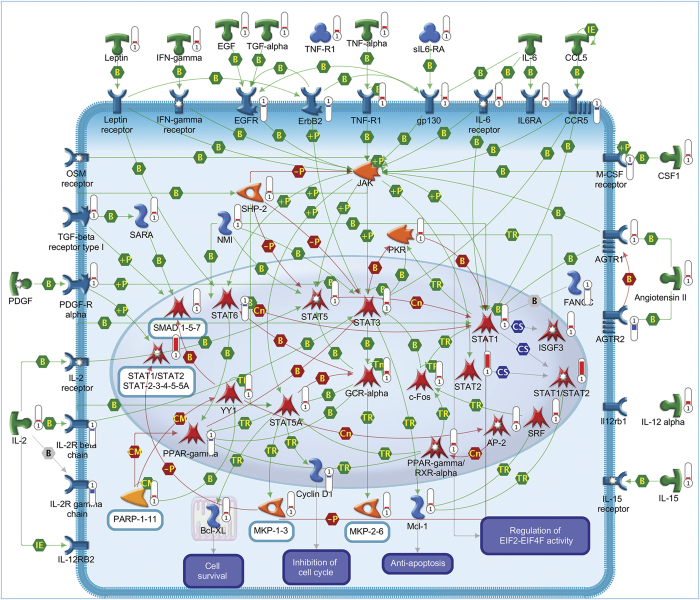
JAK-STAT (Janus kinase/signal transducers and activators of transcription) pathway. The JAK-STAT system consists of three main components: (1) a membrane receptor, (2) JAK and (3) the signal transducer and activator of transcription (STAT). It is activated by different signals, including interferon, interleukin, growth factors and other chemical messengers that activate the kinase function of JAK, which autophosphorylates itself. The STAT proteins then bind the phosphorylated receptor, where they are phosphorylated by JAK. The phosphorylated STAT proteins bind to another phosphorylated STAT protein (dimerizes) and translocate into the nucleus where promote transcription of genes responsive to STAT. The pathway is negatively regulated at multiple levels. Pathway objects and links are described separately in Supplementary Figure 16.

**Figure 6:**
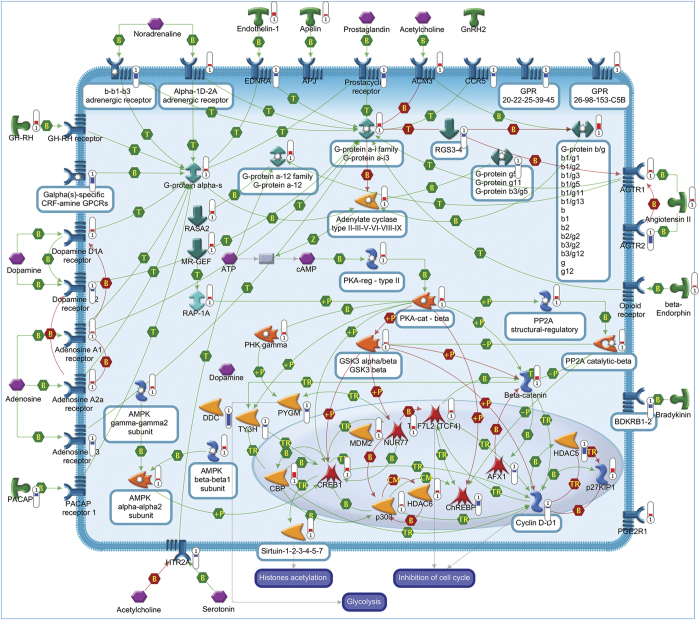
Protein kinase A (PKA) signaling pathway. The majority of G-protein coupled receptors (GPCR) are associated with activation of distinctive adenylate cyclases (ACs) to regulate intracellular cAMP levels. When active, AC produces the second messenger cAMP in response to a wide range of signal-transduction pathways. The main target of cAMP is PKA. Some of the major substrates of PKA include GSK3. PKA phosphorylates and inactivates GSK3, preventing neurodegeneration. PKA also phosphorylates the transcription factor CREB, which in turn allows the recruitment of the coactivator CREB-binding protein (CBP). Thus, PKA is important for an increasing number of physiological processes, such as regulation of the cell cycle that involves chromatin condensation and decondensation. Apart from PKA, other direct targets of cAMP include PDE, p70S6K/RPS6KB1 and PLA2. Pathway objects and links are described separately in Supplementary Figure 16.

In accordance with the crucial role of the MAPK pathway in mediating the survival effects of IGF-1 and PACAP,^1^ several members of this pathway were found overexpressed following SP treatment (Figure 2). A prominent neuroprotective role in CGNs is also played by the PKA pathway. Consistently, we observed the upregulation of different subunits of PKA in rescued CGNs, including anchoring and interacting proteins, together with different adenylate cyclases and different upstream receptors of this pathway (Figure 6). Such changes are in line with previous findings showing the ability of these proteins to promote survival in serum-deprived neurons.^22^ Upregulation of various components of the PI3K/AKT pathway also confirms the need to maintain this pathway activated to prevent neuronal apoptosis (Figure 4).^23^ The increased expression of different components of the STAT and WNT pathways provides further evidence of their involvement in promoting neuronal survival and inhibiting the death process (Figures 4 and 5).^23,24^ In agreement with the ability of different PKC isoforms to regulate apoptosis and survival,^25^ SP exerted a double activity on the PKC pathway (Figure 3).^26^

#### Ion homeostasis

Conforming with the ability of IGF-1 and PACAP to induce transcripts encoding for proteins controlling calcium homeostasis during apoptosis rescue,^1^ SP elicited the overexpression of numerous genes regulating the intracellular concentration of this ion. Upregulation of these genes, together with those encoding for proteins that mediate Ca^2+^ responses or Ca^2+^-dependent transcription, support their role in preventing cell death and neurodegeneration.^27^ Deregulated genes following SP treatment also included those encoding for different potassium channels and other transporters known to promote prosurvival effects (Figure 3).^28^

### Cell adhesion, cytoskeleton remodeling and endosomal/lysosomal pathway

#### Cell adhesion

**Figure 7:**
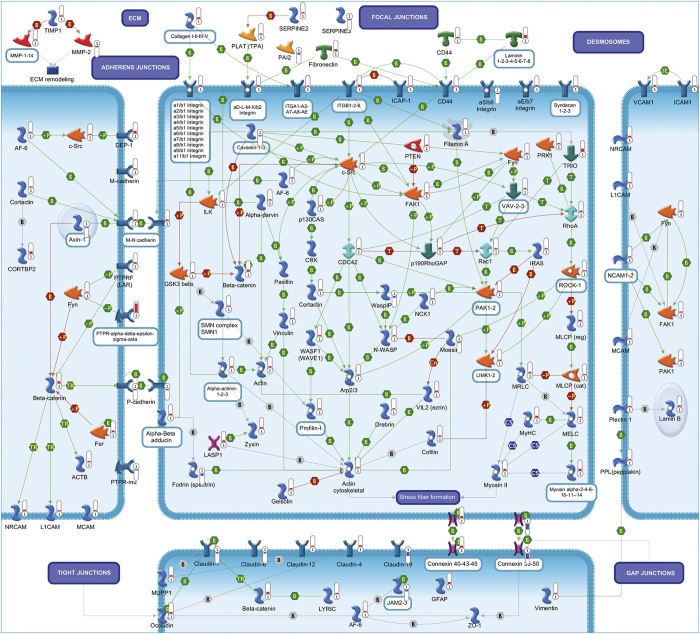
Cell adhesion. Focal adhesions are complexes through which signals are transmitted to extracellular matrix (ECM) and interacting cells. Connection between focal adhesions and ECM is realized through subcellular structures, including integrins that bind to extracellular proteins. The intracellular domain of integrin binds to the cytoskeleton via adapter proteins. Other intracellular proteins associate with this complex. Adherens junctions are subapical structures that function as mediators of cell–cell adhesion. They have a crucial role as sensors of extracellular stimuli. Cadherins are principally responsible for cell–cell adhesion. The extracellular domain of cadherin forms complexes with the extracellular domains of cadherin on neighboring cells. The cytoplasmic domain of cadherin associates with catenins, which provide anchorage to the actin cytoskeleton. Tight junctions are areas of two cells forming an impermeable barrier. They are composed of sealing strands, each of which is formed from a row of transmembrane proteins embedded in both plasma membranes, with extracellular domains joining one another directly. Although different proteins are present, the major are claudins and occludins. These associate with different membrane proteins that anchor the strands to the actin cytoskeleton. Gap junctions are transmembrane channels that connect the cytoplasm of two cells, allowing various molecules to directly pass. Each single channel comprises two hemichannels composed by connexin proteins. The cytoplasmic domain of connexins binds to ZO-1, allowing the association between the gap and tight junctions. Desmosomes are structures specialized for cell-to-cell adhesion, arranged on the sides of plasma membrane and linking surface proteins to intracellular keratin filaments. The inner dense plaque proteins are attached to intermediate filaments. Pathway objects and links are described separately in Supplementary Figure 16.

CGNs rescued by SP showed the altered expression of genes encoding for various components of focal adhesions, including 14 extracellular matrix proteins, 12 subunits of integrins and their receptors and 9 intracellular proteins. These changes are consistent with the known role of these proteins to provide a suitable substrate for neuronal survival.^29^ Similarly, the upregulation of several components of adherens junctions highlights the importance of this adhesion system in promoting neuronal survival and limiting neurodegeneration.^30^ Overexpression of genes encoding numerous components of tight and gap junctions, as well as those encoding for two desmosomal proteins, supports a possible involvement of these adhesion systems in neuronal activity and survival.^31^ We also observed the differential expression of some matrix metalloproteinases, together with the metallopeptidase inhibitor *Timp1*, which are associated with neuroprotection (Figure 7).^32^

#### Cytoskeleton remodeling

**Figure 8:**
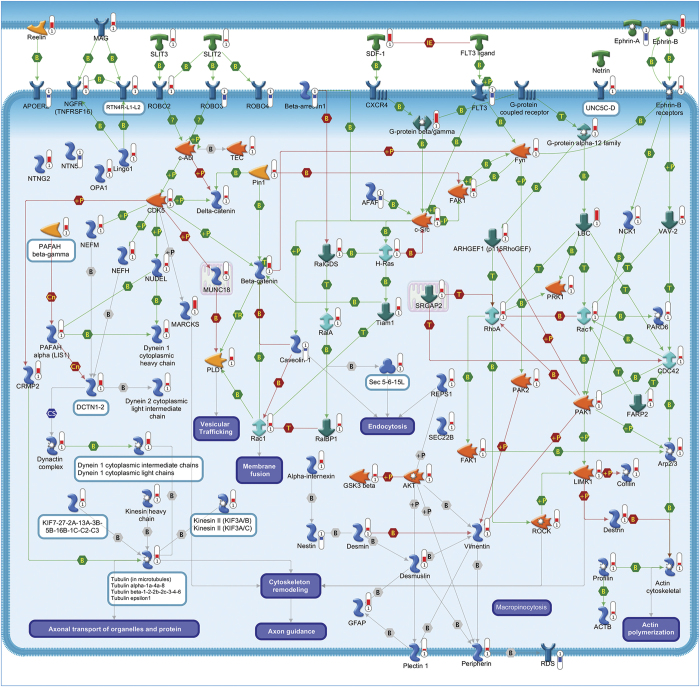
Cytoskeleton remodeling and axonal transport. The cytoskeleton consists of three distinct, yet interconnected filament systems: microfilaments (actin filaments), intermediate filaments (IFs) and microtubules. Microfilaments are the thinnest filaments of the cytoskeleton and are composed of linear polymers of actin subunits. They act as tracks for the movement of myosin molecules and are controlled by the Rho family of small GTP-binding proteins such as Rho, Rac and Cdc42. Intermediate filaments (vimentin, desmin, desmuslin, glial fibrillary acidic protein, peripherin, nestin, *α*-internexin) organize the internal tridimensional structure of the cell, anchoring organelles and serving as structural components of the nuclear lamina. Neurofilaments are the principal intermediate filament type expressed by neurons and are formed by coassembly of neurofilament light polypeptide (NEFL), neurofilament medium polypeptide (NEFM) and heavy neurofilament subunit (NEFH) subunits. Peripherin is another IF protein. Neurofilaments are important protein cargoes for actin-associated motors, such as myosin and microtubule-associated motor, such as kinesin. Microtubules are polymers of *α*- and *β*-tubulin and, in association with other proteins such as dynein and dynactin, are important for axonal transport. Ephrins and their receptor tyrosine kinases play a pivotal role in axon guidance and synaptogenesis. Ephrin-A activation leads to the actin cytoskeleton reorganization and endocytosis. Ephrin-A signaling also promotes neurite outgrowth. Ephrin receptors also maintain feedback mechanisms that reverse signaling through their ephrin ligands. Clustering of ephrin-A molecules with ephrin-A receptors increases cellular adhesion. Conversely, activation of ephrin-B1 leads to the disassembly of focal adhesions. Pathway objects and links are described separately in Supplementary Figure 16.

Changes in transcript levels of various structural and regulatory components of myosin filaments, cytoskeleton, actin-binding proteins, such as actin cytoskeletal and tubulin, support the general role of cytoskeleton in neuronal apoptosis.^33^ In addition, the altered expression of different dyneins, dynactins and kinesins supports the need of an efficient retrograde and anterograde axonal transport for ensuring neuronal survival.^34^ Differential expression of various ephrins points out the role of axonal guidance and neuronal outgrowth in neuronal vulnerability (Figure 8).^35^

#### Endosomal/lysosomal pathway

Changes in this pathway profoundly influence cell survival by regulating the expression of specific proteins on the cell surface through their internalization and lysosomal degradation. Consistent with this, CGNs rescued by SP displayed the altered expression of various endosomal–lysosomal markers, such as the endosomal GTPases Rab. This is in line with previous data showing the ability of this system to mediate neuroprotective effects.^36^ In addition, we identified the differential expression of four vesicle-associated membrane proteins and three synaptosomal-associated proteins, which are correlated with cell viability (Supplementary Figure 9).^37^

### Metabolism

#### Energy metabolism

Consistent with the rapid decrease in energy metabolism occurring in apoptotic neurons,^38^ CGNs rescued by SP showed the coordinated overexpression of transcripts for 3 glucose transporters, 11 glycolysis enzymes, 13 Krebs cycle enzymes and 29 different subunits of the oxidative phosphorylation complexes. These changes are consistent with the ability of energy to favor neuronal survival.^39^ SP neuroprotective action was also associated with the upregulation of genes encoding for 12 enzymes involved in *β*-oxidation that further reflects the need of a greater energy demand for the implementation of survival and apoptotic programs.^40^ In response to the treatment with SP, we also detected the overexpression of the cellular energy status sensor *Prkaa2* whose inhibition sensitize neuronal cells against apoptotic death (Supplementary Figure 10).^41^

### Transcriptional, translational and epigenetic regulation

#### Transcriptional regulation

In agreement with the rescue effect of IGF-1 and PACAP,^1^ SP treatment upregulates *Jun*, *Fos* and *Srf* genes, together with other prosurvival transcription factors (Figures 2, 4,5,6 and Supplementary Figure 11).^42^

#### Translation regulation

Differential expression of a remarkable number of aminoacyl-tRNA synthetases and other factors implicated in translation in rescue CGNs is compatible with the need for a correct protein synthesis during neuronal survival (Figure 4 and Supplementary Figure 11).^43^

#### Epigenetic mechanism

The increased expression of the histone acetyltransferases (HATs) and the loss of function of several histone deacetylases (HDACs) have a crucial role in neuronal survival. Upregulation of numerous HATs and other genes regulating a series of epigenetic changes supports their neuroprotective action.^44^ Increased expression of DNA methylase, histone methylase and distone demethylase transcripts reinforce the hypothesis of a possible deregulation of DNA and histone methylation during neuronal death.^45^ In addition, upregulation of some histone variants and histone kinase *Tlk2* might further contribute to the gene expression regulation in rescued CGNs (Supplementary Figure 12).

### Cell cycle

The resume of quiescent cell cycle caused by the functional alteration of a series of cell cycle regulators is a key event in neuronal apoptosis.^46^ Changes in expression of these regulators observed in rescued CGNs confirm their role in promoting cell survival and contributing to the arrest of cell cycle. At the same time, downregulation of *Cdc25b* and *Wee1* is consistent with their known role in promoting S-phase progression and triggering apoptosis.^47^ Similarly, increased expression of the CDK inhibitors supports their potential role in protecting CGNs during KCl withdrawal-induced apoptosis by inducing cell cycle arrest (Supplementary Figure 13).^48^

### Defense mechanisms

Although neurons are the most vulnerable cells to ROS increase, they possess a series of neuroprotective mechanisms based on antioxidant systems. Upregulation of genes encoding antioxidant or glutathione metabolism proteins supports the hypothesis of an increased oxidative stress and mitochondrial dysfunction in vulnerable neuronal populations and their role in protecting CGNs from oxidative stress-induced apoptosis.^49^ Increased expression of proteins involved in DNA repairing might further contribute to the protection of CGNs against cell death induced by oxidative stress (Supplementary Figure 14).

To counteract multiple signals triggering apoptosis, cells devise a large number of prosurvival strategies involving activation of different Hsps and antiapoptotic factors. CGNs rescued by SP showed the upregulation of numerous Hsps underlining the importance of these proteins in the survival mechanism induced by trophic factors (Figure 4 and Supplementary Figure 14).^50^

#### Ubiquitin-proteasome system

Defects in the ubiquitin-proteasome system profoundly influence the neuronal viability.^51^ In line with this, rescued CGNs exhibited the coordinated upregulation of different subunits of proteasome 26S complex and components of ubiquitin ligases complexes, including adaptor, scaffold and F-box proteins (Supplementary Figure 15).

## Conclusion

This work represents a glimpse of the complex transcriptional programs underlying apoptosis and its rescue by SP. In addition to elucidating the common mechanism by which neurotrophic factors mediate CGN survival, many of the genes associated with SP survival effect encode for drug targets whose modulation might exert antiapoptotic or prosurvival effects. Although the functional exploitation of these targets goes beyond the aim of the present study, we pharmacologically inhibited one of these targets, *Myc*, showing its direct involvement in the neuroprotective activity of SP. The systems biology-based perspective described here, therefore, may pave the way towards an innovative pharmacology based on targets that are downstream of neurotrophic factor receptors.

## Materials and Methods

### Materials

All the substances were obtained from Sigma-Aldrich (Milano, Italy), unless otherwise specified.

### Experimental procedure

#### Neuronal cultures

Experimental animal procedures were reviewed and approved by the Institution Committee and carried out in accordance with the European Communities Council Directive of 24 November 1986 (86/609/EEC). Primary cultures of CGNs were obtained from dissociated cerebella of 8-day-old (P8) Wistar rats (Charles River) and prepared as described previously.^1^ After 6 *days in vitro* (DIV), extracellular KCl was shifted from 25 to 5 mM for neuronal apoptosis induction. After two washes with serum-free basal medium Eagle containing 5 mM KCl, neurons were incubated with the same medium (K5) or treated with different concentrations of SP (10, 50, 100, 200 and 500 nM) or the Myc inhibitor, 10058-F4 (10, 20 *μ*M). Control neurons were grown in serum**-**free medium supplemented with 25 mM KCl (K25). Neuronal viability was assessed 48 h later by counting the number of intact nuclei according to the method described previously.^13^

#### Immunocytochemistry

Experiments were carried out as specified previously.^52^ Briefly, CGNs were fixed with 4% paraformaldehyde for 15 min and then incubated in 1% bovine serum albumin, 10% normal goat serum, 0.3 M glycine in 0.1% phosphate-buffered saline (PBS)-Tween for 1 h to permeabilize them and block nonspecific protein–protein interactions. Cells were then incubated with the primary polyclonal antibody raised against NK1 receptor (1 : 400 in PBS; Abcam, Cambridge, UK) or NeuN (1 : 200; Sigma-Aldrich) overnight at +4 °C, and then washed in PBS and incubated with a goat anti-rabbit rhodamine-conjugated secondary antibody (1 : 1000; Sigma-Aldrich) for 30 min at room temperature. Cells were also stained with Hoechst 33258 (250 ng/ml) for 5 min at room temperature to label neuronal nuclei. Neurons were then visualized by fluorescent microscopy.^52^

#### Microarray experiments and data analysis

Whole**-**genome expression analysis was performed in serum-deprived cells 6 h after the induction of neuronal apoptosis and treatments with the maximal effective dose of SP (200 nM). RNA extraction, integrity, labeling and hybridization were performed following the protocols outlined by the manufacturers as described previously.^1^ Microarrays were scanned at 5-μm resolution using a GenePix Personal 4100A Microarray Scanner and the GenePix Pro 6.0 Acquisition and Data-Extraction Software (Molecular Devices, Sunnyvale, CA, USA). Raw data were processed and analyzed with GeneSpring GX 13 (Agilent Technologies, Milano, Italy). To remove unreliable data, all genes from all samples were filtered for quality to include only probe data fulfilling all of the following criteria in all replicates of at least one out of four experimental conditions: the spot had <3% of saturated pixels at 532 nm; the spot was not flagged ‘bad’, ‘not found’ or ‘absent’; the spot was detectable well above background (signal-to-noise ratios at 532 nm >10). Data filtering listed 29 892 probes out of a total of 41 012 present on the microarray. Raw data are available in gene expression omnibus database with the accession number GSE67788. Genes in our quality-filtered data set were screened by a one-way ANOVA using Welch's *t*-test, followed by the Benjamin and Hochberg false discovery rate (FDR) procedure as a multiple testing correction and the Tukey’s *post hoc* test. Genes with a corrected *P*-value <0.05 were selected as differentially expressed genes. To analyze gene expression changes in the context of known biological pathways, we used MetaCore (Thomson Reuters, Milano, Italy). *P*-values were calculated using a basic formula for the hypergeometric distribution, where the *P*-value essentially represents the probability of a particular pathway arising by chance. To limit the possible number of type I errors among the results, an FDR threshold of 0.05 was used to identify statistically significant pathways. To unravel the genetic networks involved, reduce redundancy of deregulated pathways and simplify their comprehension, the most significant variations were summarized in hand-curated figures drawn by Pathway Map Creator (Thomson Reuters, Milano, Italy). Statistically significant genes and pathways are listed in Supplementary Data S9.

#### Quantitative real-time reverse transcription-PCR

Although our data represent the average gene expression from four replicates, we confirmed the reliability of the microarray data by performing quantitative real-time RT-PCR according to the protocol described previously^1^ and using the primers listed in Supplementary Table 1.
