# Supplementary material for: Transcriptional landscapes at the intersection of neuronal apoptosis and substance P-induced survival: exploring pathways and drug targets
Source: Cell Death Discov. 2016 Aug 1;2:16050–. doi: 10.1038/cddiscovery.2016.50 (PMC4979452; doi:10.1038/cddiscovery.2016.50)
Supplement: Supplementary Table 1 [file cddiscovery201650-s1.pdf]

Table 1. Validation of microarray data by real-time quantitative RT-PCR.

Name	Genbank		K25	K5	K5 + SP	Forward primer	Reverse primer
**Early growth response protein 1 (Egr1)**	U75397	Microarray	-0,94	0,11	1,58	GTTGGAATGCTGTGGTTACC	GCCAAACAAGTCACTTTGTTTA
		RT-PCR	1475 ± 85	3019 ± 108	3361 ± 102		
**NIPA-like domain containing 2 (Nipal2)**	NM_001130559	Microarray	-1,26	-0,22	0,71	ACATGGAGAAGCAACCTCTG	CTCCGTAATTGTCAGCAGCT
		RT-PCR	667 ± 16	2011 ± 91	4065 ± 133		
**Family with sequence similarity 171, member A2 (Fam171a2)**	XM_001081512	Microarray	0,11	-0,63	-1,58	AGGACAACGTGTACCGCAAT	TGGGGATCAGGTTGAGGGAA
		RT-PCR	2877 ± 128	1136 ± 92	871 ± 65		
**DEAD (Asp-Glu-Ala-Asp) box helicase 56 (Ddx56)**	NM_0010042112	Microarray	0,35	-0,53	0,18	TCTTAGGCTGTCACCGACTT	ATTAGCCACTCTCACATCGC
		RT-PCR	2493 ± 106	163 ± 12	2166 ± 77		
**Zinc finger protein 423 (Zfp423)**	XM_001081512	Microarray	-1,23	-0,27	0,59	GAAGACAGGAACAGCGTGAC	GTCGTCATCACCATCTCCAG
		RT-PCR	277 ± 31	856 ± 35	3184 ± 69		
**Neuronal pentraxin I (Nptx1)**	NM_153735	Microarray	-1,50	-0,39	1,14	GGAGCTGAATGGTTACATGG	ATAAGTCCACTGCGCACAGA
		RT-PCR	781 ± 32	2630 ± 85	4502 ± 181		

Microarray: mean normalized value (Log scale)
Quantitative RT-PCR: mean ± SEM of copies/100 pg RT-RNA
