# Supplementary figures and images for: Transcriptional landscapes at the intersection of neuronal apoptosis and substance P-induced survival: exploring pathways and drug targets
Source: Cell Death Discov. 2016 Aug 1;2:16050–. doi: 10.1038/cddiscovery.2016.50 (PMC4979452; doi:10.1038/cddiscovery.2016.50)

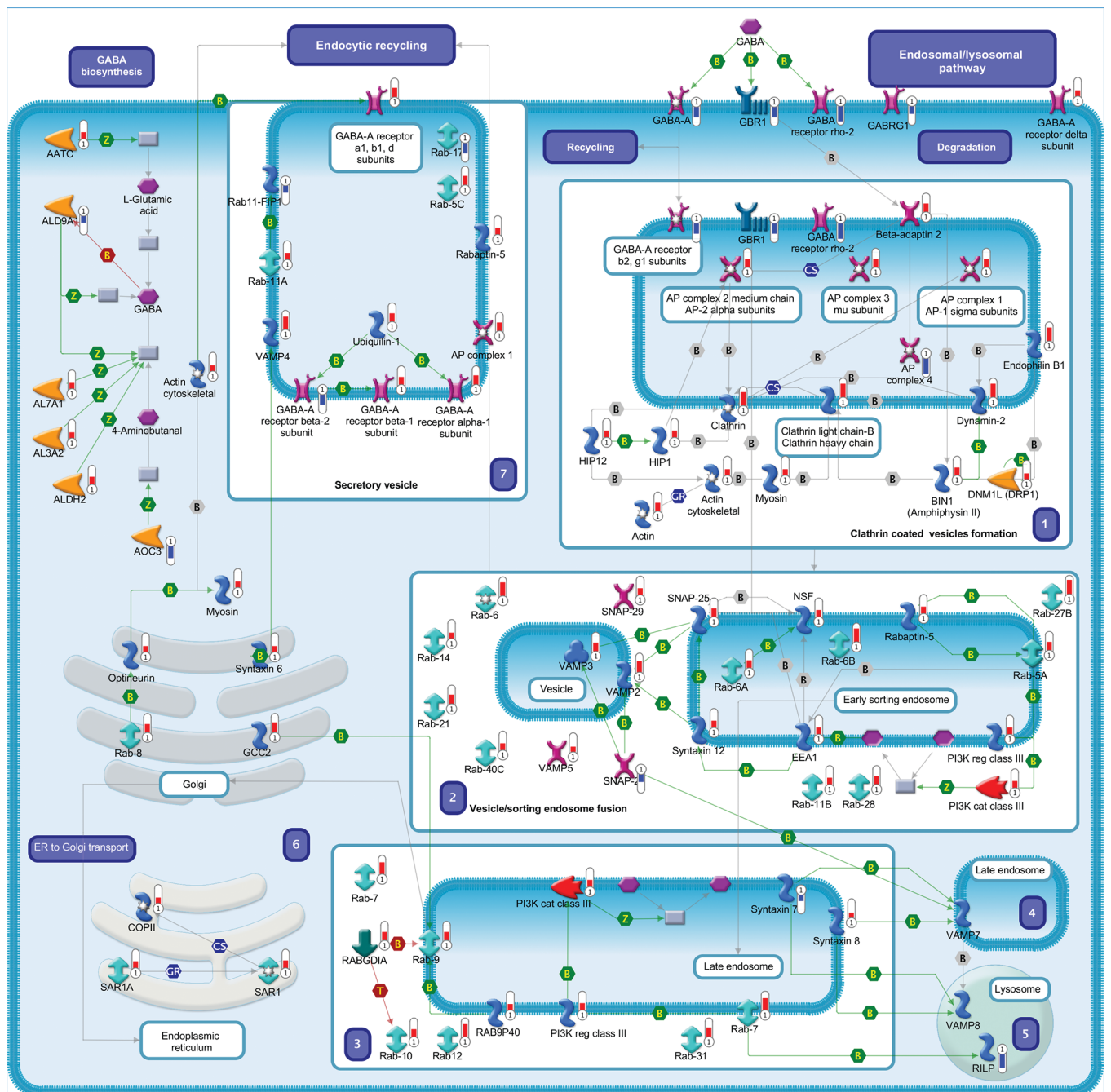

Supplement: Supplementary Figure 1 [file cddiscovery201650-s2.pdf]

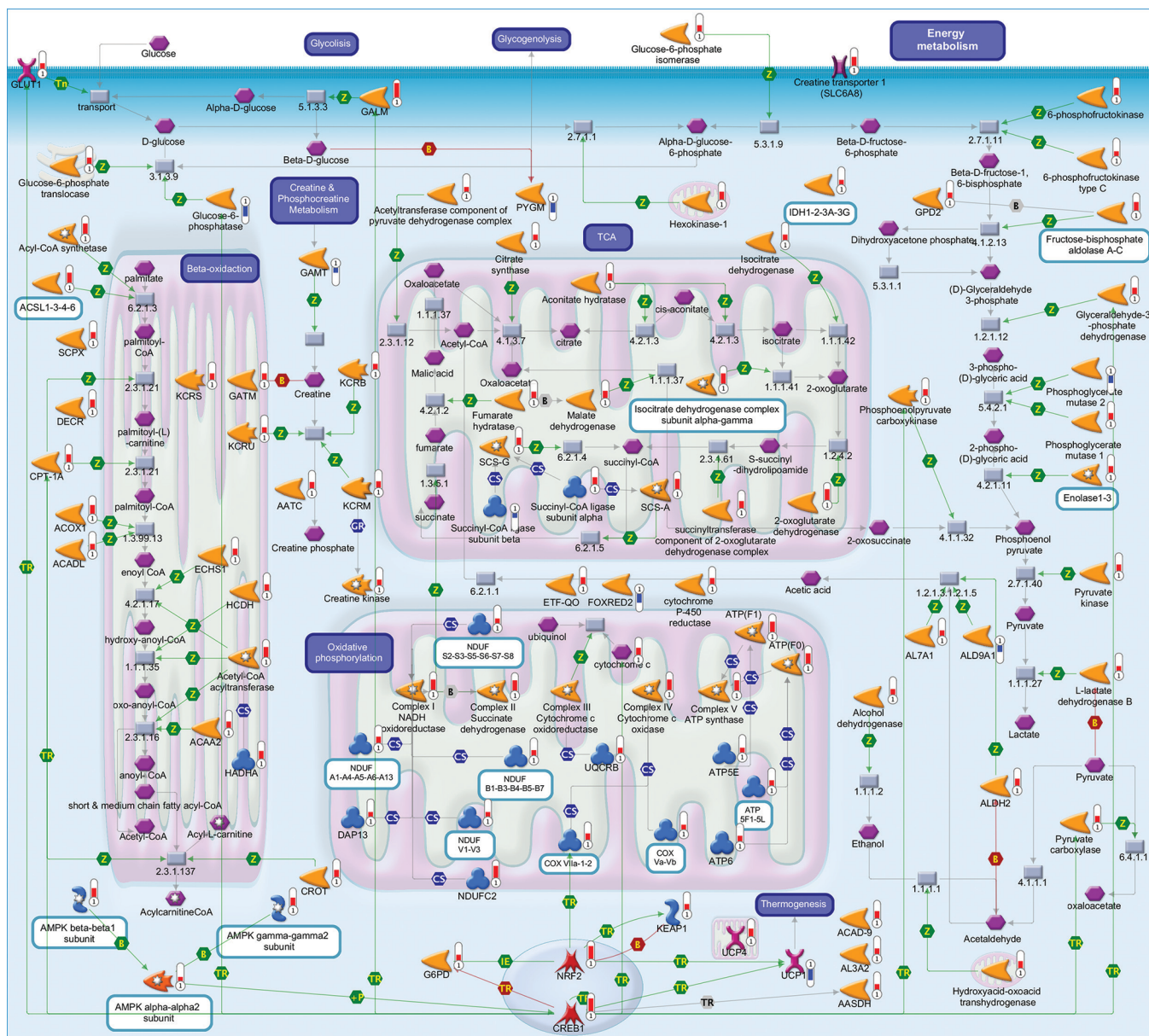

Supplement: Supplementary Figure 2 [file cddiscovery201650-s3.pdf]

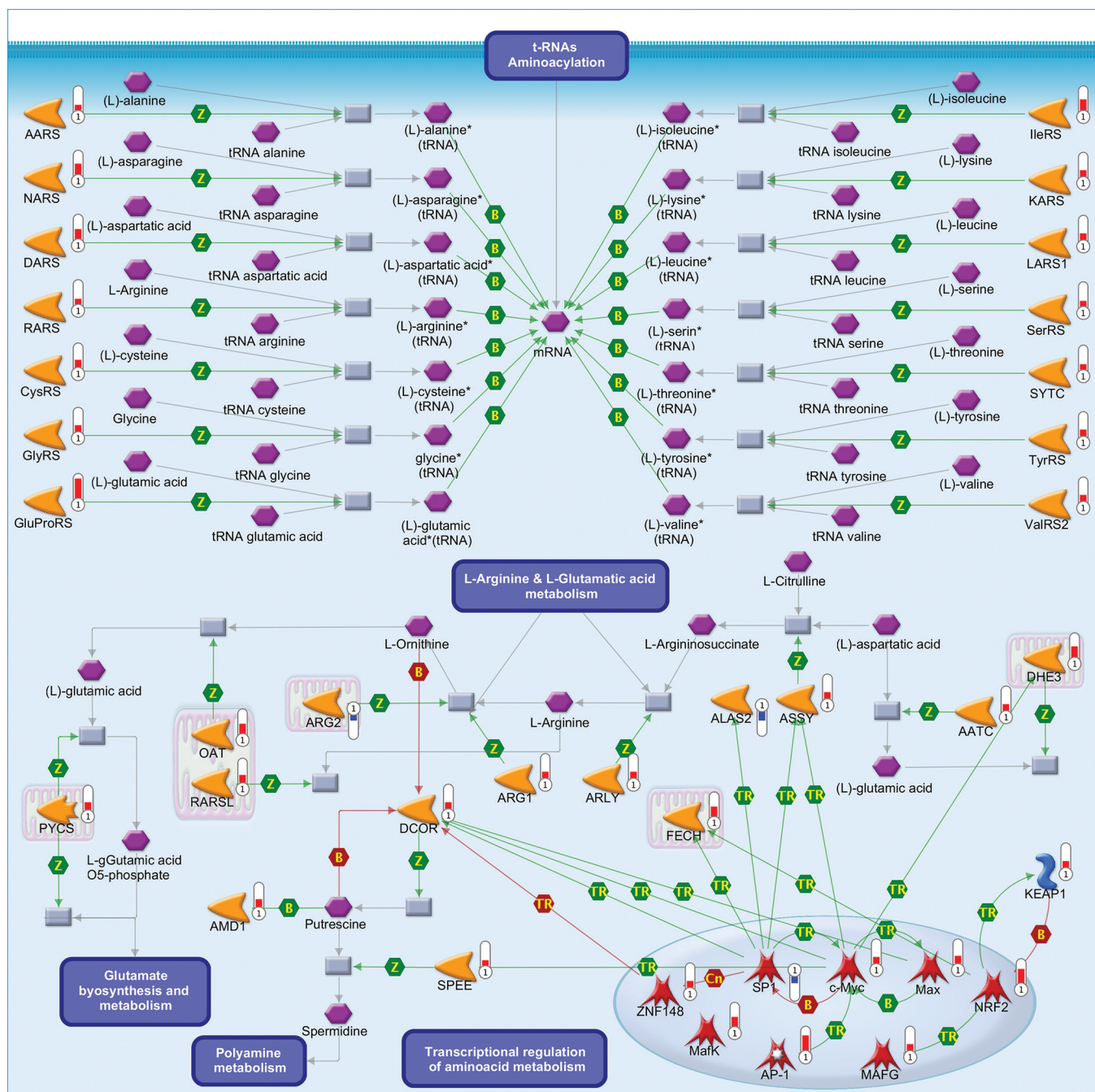

Supplement: Supplementary Figure 3 [file cddiscovery201650-s4.pdf]

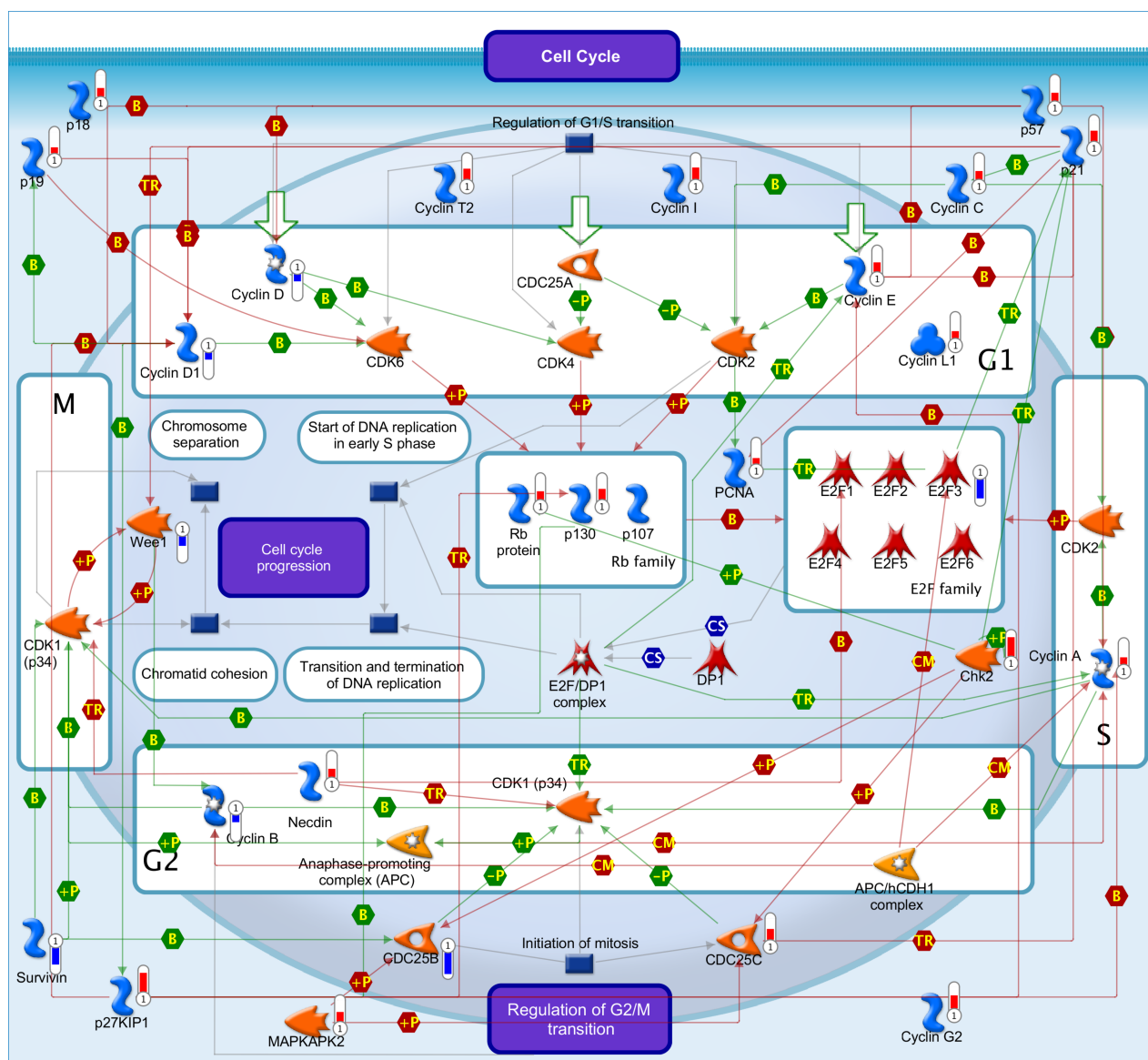

Supplement: Supplementary Figure 5 [file cddiscovery201650-s6.pdf]

## Defense mechanisms

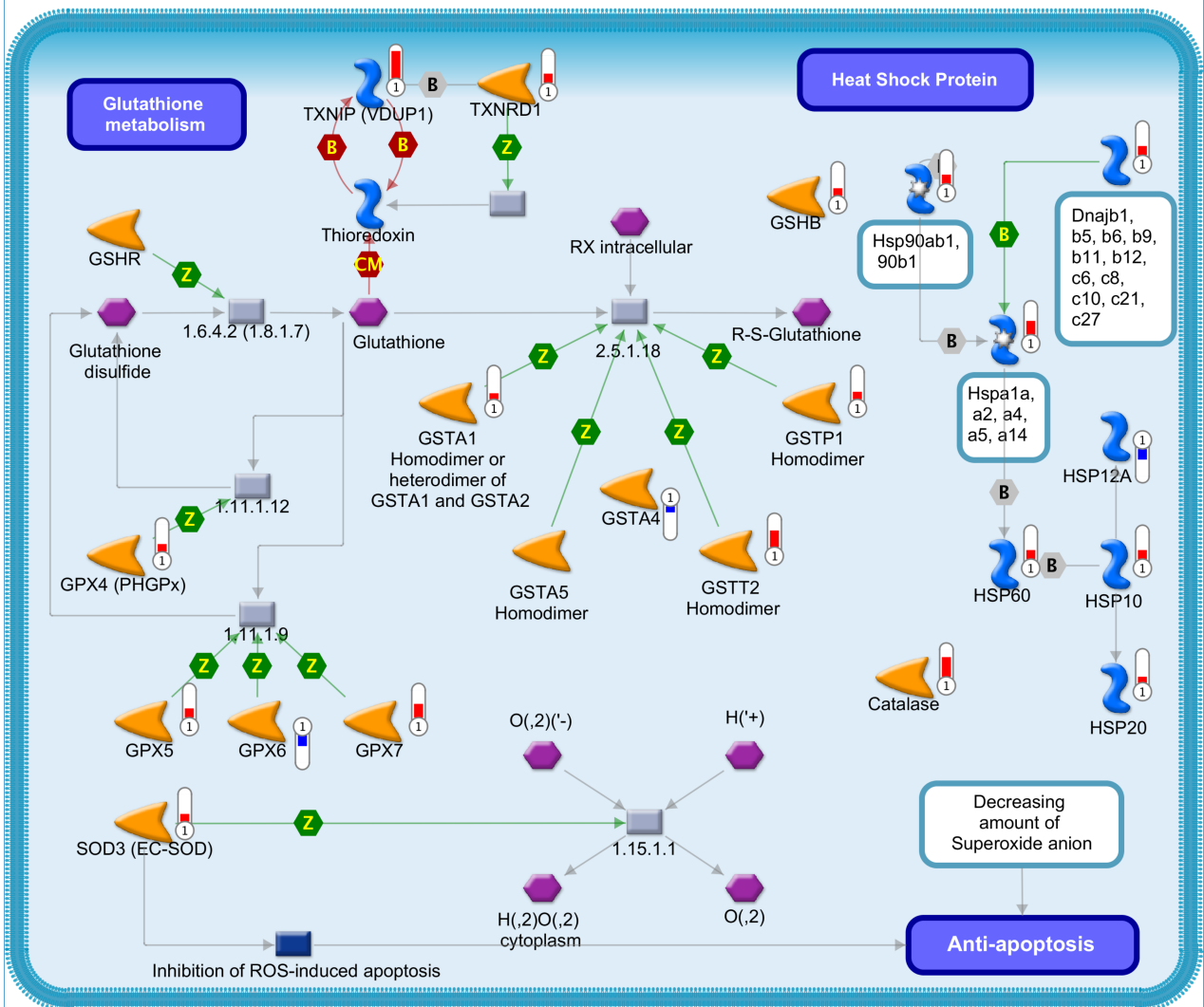

Supplement: Supplementary Figure 6 [file cddiscovery201650-s7.pdf]

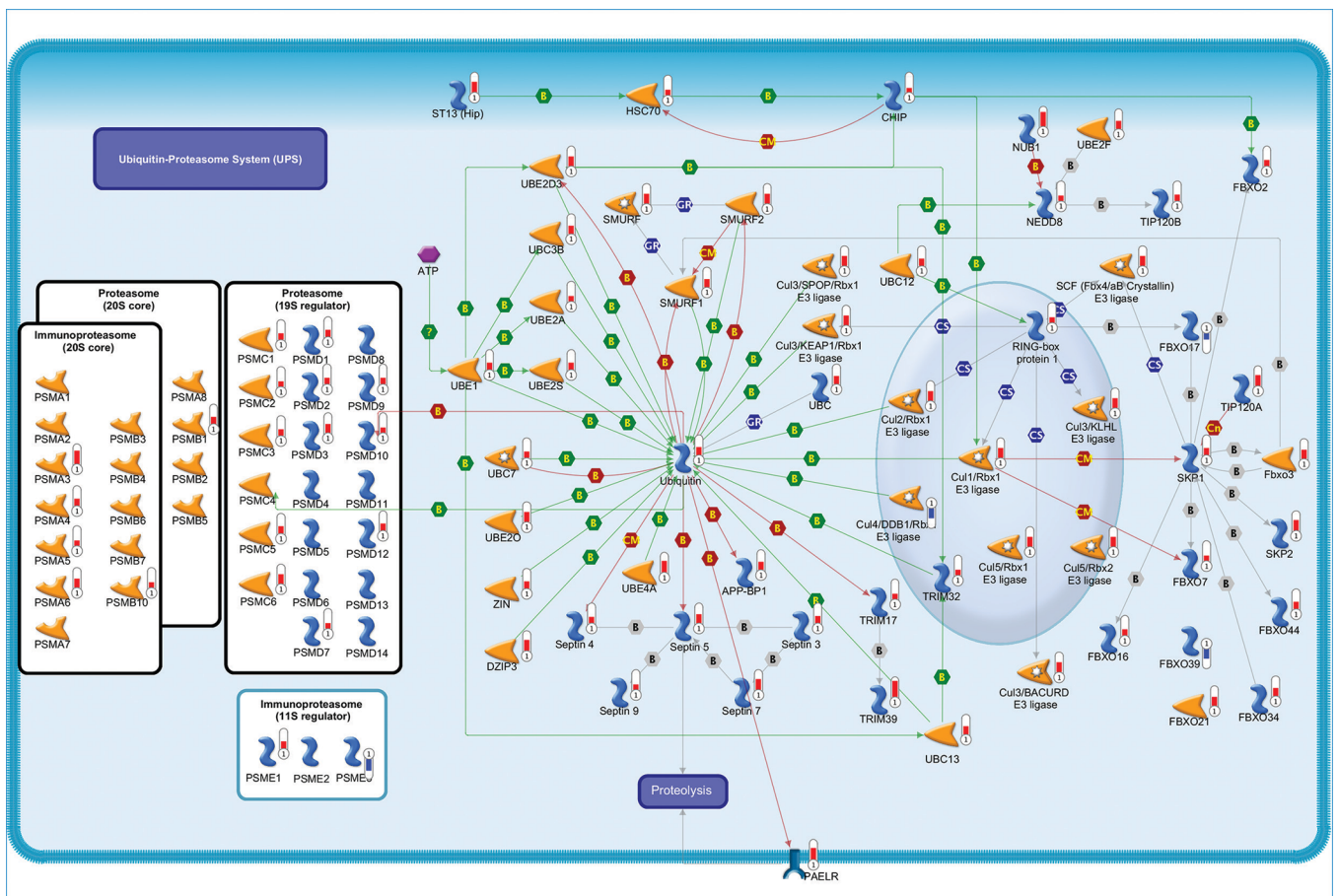

Supplement: Supplementary Figure 7 [file cddiscovery201650-s8.pdf]

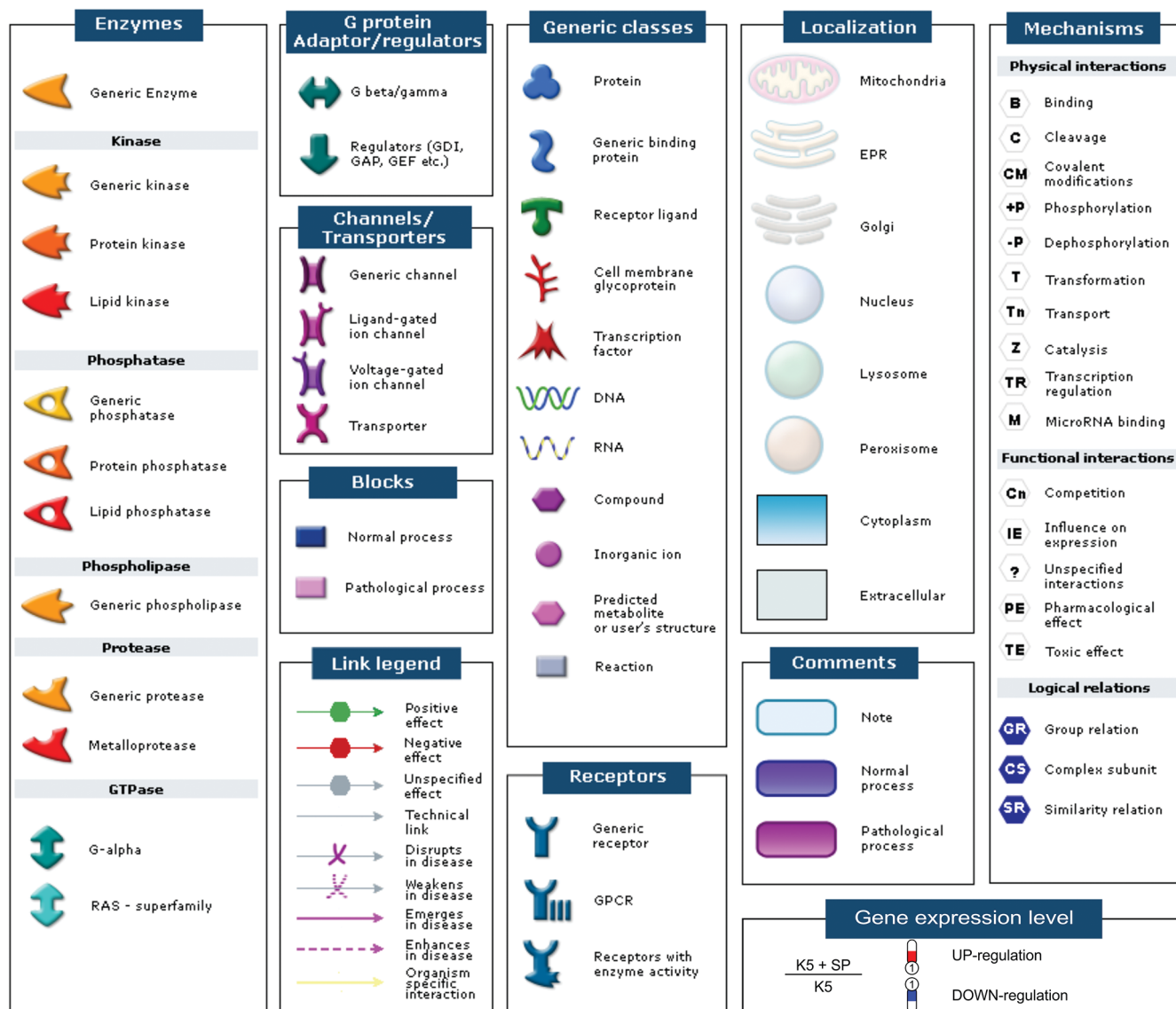

Supplement: Supplementary Figure 8 [file cddiscovery201650-s9.pdf]
