# Supplementary material for: Transcriptional landscapes at the intersection of neuronal apoptosis and substance P-induced survival: exploring pathways and drug targets
Source: Cell Death Discov. 2016 Aug 1;2:16050–. doi: 10.1038/cddiscovery.2016.50 (PMC4979452; doi:10.1038/cddiscovery.2016.50)
Supplement: Supplementary Figure 4 [file cddiscovery201650-s5.pdf]

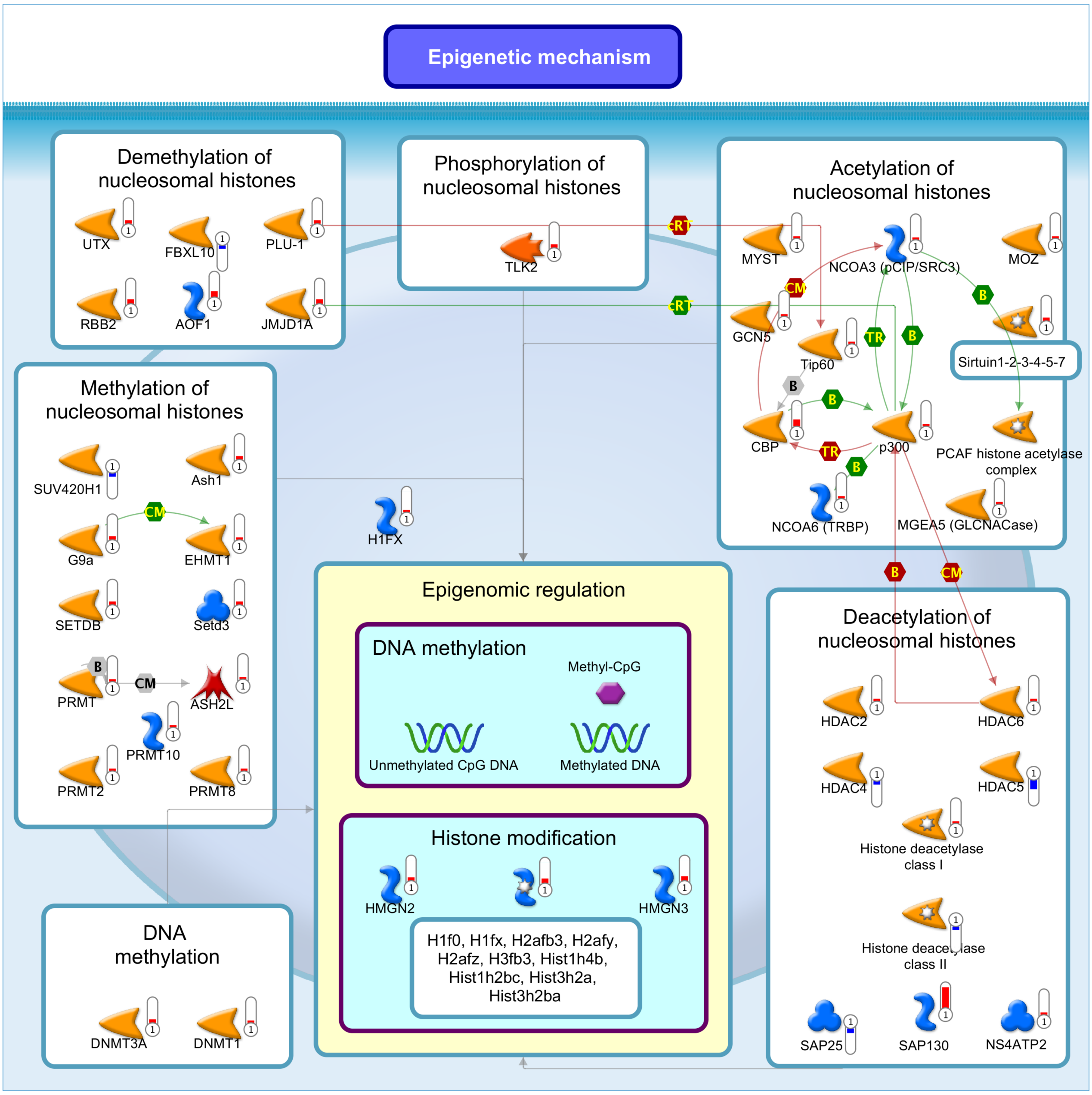

Epigenetic mechanism
Demethylation of nucleosomal histones
UTX
FBXL10
PLU-1
RBB2
AOF1
JMJD1A
Phosphorylation of nucleosomal histones
TLK2
Acetylation of nucleosomal histones
MYST
NCOA3 (pCIP/SRC3)
MOZ
GCN5
Tip60
Sirtuin1-2-3-4-5-7
CBP
p300
PCAF histone acetylase complex
NCOA6 (TRBP)
MGEA5 (GLCNACase)
Methylation of nucleosomal histones
SUV420H1
Ash1
G9a
EHMT1
SETDB
Setd3
PRMT
ASH2L
PRMT10
PRMT2
PRMT8
H1FX
Epigenomic regulation
DNA methylation
Methyl-CpG
Unmethylated CpG DNA
Methylated DNA
Histone modification
HMGN2
HMGN3
H1f0, H1fx, H2afb3, H2afy, H2afz, H3fb3, Hist1h4b, Hist1h2bc, Hist3h2a, Hist3h2ba
DNA methylation
DNMT3A
DNMT1
Deacetylation of nucleosomal histones
HDAC2
HDAC6
HDAC4
HDAC5
Histone deacetylase class I
Histone deacetylase class II
SAP25
SAP130
NS4ATP2
